# Bioactive Phenolics of *Hyoscyamus muticus* L. Subsp. Falezlez: A Molecular and Biochemical Approach to Antioxidant and Urease Inhibitory Activities

**DOI:** 10.3390/ijms26010370

**Published:** 2025-01-04

**Authors:** Sabrina Lekmine, Ouided Benslama, Bachir Bensalah, Nabil Touzout, Hamza Moussa, Hichem Tahraoui, Mohammad Shamsul Ola, Haroun Hafsa, Jie Zhang, Abdeltif Amrane

**Affiliations:** 1 Biotechnology, Water, Environment and Health Laboratory, Abbes Laghrour University, Khenchela 40000, Algeria; 2 Laboratory of Natural Substances, Biomolecules, and Biotechnological Applications, Department of Natural and Life Sciences, Larbi Ben M’Hidi University, Oum El Bouaghi 04000, Algeria; 3 Department of Biology, Faculty of Natural and Life Sciences, University of Ghardaia, Ghardaia 47000, Algeria; 4 Laboratory of Biomaterials and Transport Phenomena (LBMTP), University Yahia Fares, Médéa 26000, Algeria; 5 Laboratoire de Gestion et Valorisation des Ressources Naturelles et Assurance Qualité (LGVRNAQ), Faculté des Sciences de la Nature et de la Vie et des Sciences de la Terre, Université de Bouira, Bouira 10000, Algeria; 6 Département des Sciences Biologiques, Faculté des Sciences de la Nature et de la Vie et des Sciences de la Terre, Université de Bouira, Bouira 10000, Algeria; 7 Laboratoire de Génie des Procédés Chimiques, Département de Génie des Procédés, Faculté de Technologie, Université Ferhat Abbas, Sétif-1, Sétif 19000, Algeria; 8 Ecole Nationale Supérieure de Chimie de Rennes, University of Rennes, CNRS, ISCR—UMR6226, 35000 Rennes, France; 9 Department of Biochemistry, College of Science, King Saud University, Riyadh 11451, Saudi Arabia; 10 Laboratory of Reaction Engineering, USTHB, BP 32, Algiers 16111, Algeria; 11 School of Engineering, Merz Court, Newcastle University, Newcastle upon Tyne NE1 7RU, UK

**Keywords:** *Hyoscyamus muticus*, LC-ESI-MS/MS, phenolic compounds, antioxidant activity, urease inhibition, molecular docking

## Abstract

This study examines the chemical composition, antioxidant properties, and urease inhibitory effects of *Hyoscyamus muticus* L. subsp. falezlez (Coss.) Maire. Using LC-ESI-MS/MS, 19 distinct phenolic compounds were identified, with chlorogenic acid being the most abundant. The ethanol extract demonstrated notable antioxidant activity, highlighting its potential for therapeutic use. Urease inhibition assays revealed a remarkable 91.35% inhibition by the *H. muticus* extract, with an IC_50_ value of 5.6 ± 1.20 μg/mL, indicating its promising role in addressing conditions linked to urease activity. Molecular docking studies further investigated the interaction between *H. muticus* phenolic compounds and urease, identifying hyperoside as a leading candidate, with a binding energy of −7.9 kcal/mol. Other compounds, such as rutin, luteolin, apigenin, kaempferol, hesperetin, chlorogenic acid, and rosmarinic acid, also demonstrated significant binding affinities, suggesting their potential to disrupt urease function. These findings highlight the therapeutic potential of *H. muticus* as a source of natural bioactive compounds, offering promising avenues for the development of novel treatments for urease-related disorders and oxidative stress.

## 1. Introduction

*Hyoscyamus muticus* L. subsp. falezlez (Coss.), commonly known as Egyptian henbane, belongs to the Solanaceae family, a group of plants renowned for their medicinal properties [1]. This subspecies is native to North Africa, particularly Algeria, where it thrives in arid and semi-arid regions [2] Traditionally, *Hyoscyamus* species have been employed in herbal medicine for their diverse pharmacological activities, including antispasmodic, analgesic, and sedative properties [3,4,5]. These attributes are attributed to the plant’s bioactive compounds, particularly alkaloids, flavonoids, and phenolic constituents, which exhibit potent biological effects [6,7].

One of the significant health challenges addressed by natural products like *Hyoscyamus muticus* is the inhibition of urease, a nickel-dependent metalloenzyme produced by a wide range of bacteria, fungi, and plants [8]. Urease plays a crucial role in the survival of microorganisms in acidic environments, particularly in the human stomach [9]. It catalyzes the hydrolysis of urea into ammonia and carbon dioxide, which results in a local increase in pH, neutralizing gastric acidity and creating a favorable environment for bacterial colonization and persistence [9,10]. Among the most notorious urease-producing bacteria is *Helicobacter pylori*, a pathogen responsible for numerous gastrointestinal disorders, including gastritis, peptic ulcers, and, in some cases, gastric cancer [11].

The overproduction of ammonia as a byproduct of urease activity is not only harmful to the gastric mucosa but also contributes to systemic health issues [8]. Ammonia is a cytotoxic compound that disrupts cellular homeostasis, leading to the generation of reactive oxygen species (ROS) and inducing oxidative stress [11]. This oxidative stress exacerbates tissue damage and promotes inflammation, further impairing the host’s ability to counteract infections [11]. Moreover, urease activity has been implicated in the formation of urinary and kidney stones, as the enzyme facilitates the precipitation of calcium and magnesium ammonium phosphate crystals in the urinary tract [12]. This condition, known as struvite stone formation, is a common complication of urease-positive bacterial infections in the urinary system [13].

In addition to its role in gastrointestinal and urinary disorders, urease is linked to hepatic encephalopathy, a severe neurological condition associated with liver dysfunction [14]. The excessive production of ammonia due to urease activity contributes to hyperammonemia, which crosses the blood–brain barrier and disrupts neuronal function, leading to cognitive impairments and confusion [15]. These diverse pathological effects highlight the importance of developing effective urease inhibitors as therapeutic agents.

While synthetic urease inhibitors, such as acetohydroxamic acid (AHA), have shown efficacy in clinical settings, their use is often limited by adverse effects, including hepatotoxicity and gastrointestinal discomfort [16]. This has driven the search for safer and more effective alternatives derived from natural sources. Plants, with their vast repertoire of bioactive compounds, represent a promising avenue for discovering novel urease inhibitors with lower toxicity and improved therapeutic profiles.

The dual role of urease in promoting bacterial survival and contributing to multiple health disorders highlights the enzyme as a critical target in the development of therapeutic strategies [17]. By disrupting urease activity, it is possible to impair the pathogenicity of bacteria such as *H. pylori*, reduce ammonia-mediated toxicity, and mitigate the progression of urease-related complications [18].

In addition to urease inhibition, antioxidant activity plays a vital role in mitigating oxidative stress, which is a key factor in the development of various gastrointestinal diseases, including gastric ulcers and cancer [19]. Reactive oxygen species (ROS), which include free radicals and peroxides, are produced as byproducts of normal cellular metabolism. However, their excessive accumulation leads to oxidative damage of cellular components, such as lipids, proteins, and DNA, contributing to the pathogenesis of chronic conditions [20] In the context of gastric health, ROS exacerbate tissue damage and inflammation, providing a conducive environment for bacterial colonization, particularly by urease-producing pathogens like *Helicobacter pylori*.

Antioxidants neutralize these harmful ROS, preventing or slowing down oxidative damage. Studies have shown that natural compounds with antioxidant properties, such as polyphenols, flavonoids, and phenolic acids, possess significant therapeutic potential. These compounds are known to scavenge free radicals and enhance the body’s own defense mechanisms, leading to improved cellular integrity and function [21].

Moreover, combining urease-inhibitory and antioxidant properties in a single compound or therapeutic approach could offer a dual advantage in treating conditions related to bacterial infections and oxidative stress. By inhibiting urease activity, these compounds can disrupt the bacterial survival mechanisms, while simultaneously reducing the oxidative burden on the tissues. This dual action would be particularly valuable in treating conditions like *H. pylori*-induced gastritis and peptic ulcers, where both bacterial virulence and oxidative stress play pivotal roles in disease progression [22].

This study focuses on *Hyoscyamus muticus* L. subsp. falezlez, with the aim of evaluating its potential as a natural urease inhibitor and antioxidant agent. The first step involves analyzing the chemical composition of the plant, with a particular focus on its phenolic content, known for its bioactivity. To assess its antioxidant properties, an in vitro antioxidant assay was performed. In addition, an in vitro anti-urease assay was conducted to investigate the plant’s ability to inhibit urease activity. Finally, molecular docking studies were carried out to explore the interactions between urease and the identified phenolic compounds at the molecular level. By combining phytochemical analysis, experimental assays, and computational methods, this study aims to contribute to the identification of effective, low-toxicity natural compounds for combating urease-associated infections and oxidative stress-related conditions.

## 2. Results

### 2.1. Survey and Measurement of Phenolic Compounds

**Figure 1:**
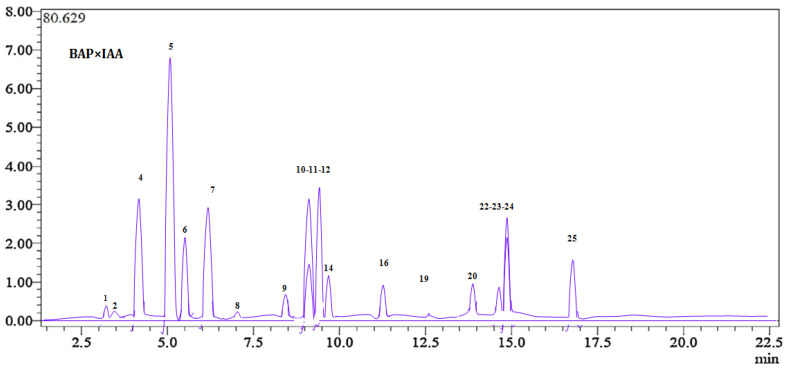
Comprehensive LC-MS/MS chromatographic visualization of *H. muticus* extract.

The comprehensive LC-ESI-MS/MS analytical technique applied to the *H. muticus* extract yielded significant and noteworthy results, as illustrated in Figure 1 and Table 1. The study confirmed the existence of 19 phenolic molecules, with the highest amounts found in chlorogenic acid (17,108.3± 1.3 µg/g E). Moreover, gallic acid (125.25 ± 3.4 µg/g E), rutin (269.25 ± 1.3 µg/g E), rosmarinic acid (125.2 ± 1.1 µg/g E), and *p*-coumaric acid (875 ± 1.3 µg/g E) were found in high concentrations. Interestingly, there was no statistically significant difference between these two molecules. Compounds with moderate quantities, such as protocatechuic acid and apigenin, were also discovered, with values ranging from 1108.3± 1.2 to 563 ± 2.3 µg/g extract.

### 2.2. Antioxidant Activity

The free radical-scavenging capacity of the *H. muticus* extract was assessed through a variety of assays, as presented in Table 2. In the CUPRAC assay, the extract demonstrated an A_0.5_ value of 22.57 ± 1.2, indicative of its considerable antioxidant capacity. Similarly, in the reducing power assay, the extract exhibited an A_0.5_ value of 13.5 ± 2.3, further underscoring its ability to reduce oxidized compounds. Notably, in the β-carotene assay, the extract displayed a notable IC_50_ value of 6.12 ± 1.8, indicating its efficacy in preventing β-carotene oxidation. Moreover, in the DMSO alkaline assay, the extract exhibited an IC_50_ value of 12 ± 1.2, suggesting its capacity to scavenge free radicals effectively. In the SNP assay, the extract demonstrated an IC_50_ value of 6.5 ± 1.5. Furthermore, in the phenonthroline assay, the extract showed an A_0.5_ value of 23 ± 1.8, highlighting its chelating activity against metal ions. Lastly, in the hydroxyl radical assay, the extract displayed an IC_50_ value of 39.95 ± 2.3, indicating its potency in scavenging hydroxyl radicals. Overall, these results elucidate the robust antioxidant potential of *H. muticus* extract across a spectrum of assays, affirming its significance as a natural source of antioxidants.

### 2.3. Urease Inhibition

The results presented in Table 3 demonstrate the urease inhibitory activity of the *H. muticus* extract in comparison to thiourea, which served as a positive control. At a concentration of 5 mg/mL, the *H. muticus* extract exhibited a substantial urease inhibition of 91.35%, indicating its strong potential as an effective urease inhibitor. The calculated IC_50_ value for the extract was determined to be 5.6 ± 1.20 µg/mL. This value suggests that *H. muticus* is relatively effective in inhibiting urease, though its activity is somewhat less potent than that of thiourea, which achieved a 96% inhibition at a much lower concentration of 5 mg/mL.

Both the *H. muticus* extract and the thiourea exhibit demonstrated considerable efficacy in inhibiting urease activity, with *H. muticus* showing a noteworthy inhibition rate and a competitive IC_50_ value. These results suggest *that H. muticus* could be a valuable candidate for further development in addressing conditions related to urease activity.

### 2.4. Molecular Docking Studies

**Figure 2:**
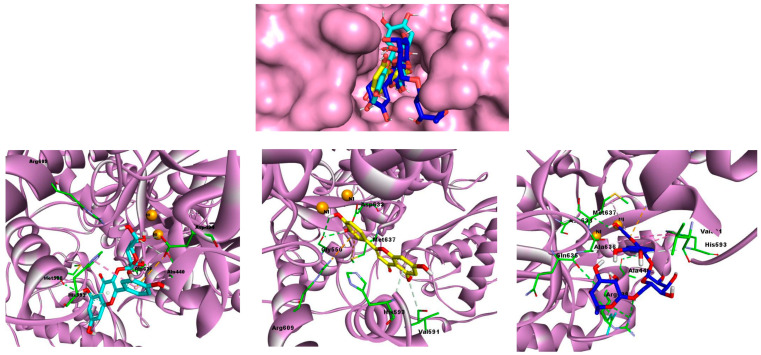
Visualization of the urease binding pocket, highlighting the docking positions of the three most efficacious phenolic compounds at the center. Surrounding graphics depict docking results for these compounds. The urease protein is shown in pink, with interacting residues in green. The phytocompounds hyperoside, luteolin, and rutin are represented in cyan, yellow, and blue, respectively.

The docking results presented in Table 4 elucidate the interaction between various phenolic compounds from *H. muticus* and urease (4H9M), providing insights into their potential as urease inhibitors. The docking conformations of the most effective compounds (hyperoside, rutin, and luteolin) within the urease binding site, alongside their respective interaction profiles, are visually represented in Figure 2. This illustration clarifies the phenolic molecules’ potential as urease activity inhibitors by providing important insights into their spatial orientation and structure of interactions within the urease binding pocket.

## 3. Discussion

Desert plants are well known for their ability to accumulate phenolic compounds, enabling them to survive in extreme arid conditions [23]. These challenging environments expose plants to cellular damage, prompting them to produce antioxidant compounds as a natural defense mechanism. Among these, phenolic compounds play a key role in protecting plant cells from environmental stress [23].

*Hyoscyamus muticus* thrives in such arid regions, where water is scarce and rainfall minimal. Our analysis identified several important phenolic compounds in this plant, including rosmarinic acid, chlorogenic acid, benzoic acid, and rutin, which contribute significantly to its antioxidant capacity. Additionally, the presence of tr-caffeic acid and p-coumaric acid, a member of the 4-hydroxycinnamic acid family, highlights the biochemical strategies *H. muticus* employs to mitigate environmental stress [24]. These compounds are known to absorb harmful high-energy radiation and convert it into safer, longer-wavelength light, thereby protecting cellular structures from damage [25].

Our findings, illustrated in Figure 1, align with previous research, particularly the work of Elsharkawy et al. (2018) [26], who used GC-MS to analyze the phenolic profile of *H. muticus* aerial parts. This study identified a variety of phenolic compounds, including ferulic acid and 4′-hydroxy-3′-methylacetophenone. Most of these compounds were found in esterified forms, with ferulic acid being the exception, appearing in its free form.

Previous studies have highlighted the significant influence of the extraction solvent on the antioxidant activity and polyphenolic content of plant extracts [27]. The choice of solvent greatly affects the efficiency of the extraction process, which in turn impacts the antioxidant yield. Antioxidants are crucial in preventing oxidative damage to lipids and protecting cells from the harmful effects of free radicals.

In the β-carotene bleaching assay, *H. muticus* extract demonstrated a remarkable antioxidant capacity, with a half-maximal inhibitory concentration (IC_50_) of 6.12 ± 1.8 µg/mL, outperforming the standard antioxidant BHT, which had an IC_50_ of 9.21 ± 0.6 µg/mL. As shown in Figure 1 and Figure 2, the extract is particularly rich in chlorogenic acid, with an impressive concentration of 17,108.3 ± 1.3 µg/g of dry extract. This high level of bioactive compounds highlights the strong antioxidant potential of *H. muticus* and its suitability as a source of natural antioxidant agents.

Our antioxidant findings resonate with previous research on *H. muticus* specimens thriving in the arid expanses of the Arabian Peninsula and the deserts of the Middle East. Elsharkawy et al. (2018) [26] investigated the ethanol extract derived from the aerial parts of *H. muticus* indigenous to Saudi Arabia’s arid zones, revealing significant antioxidant activity, with an IC_50_ value of 8.1 ± 0.65 mg/mL and an EC50 value of 12.74 ± 1.12 mg/mL. Likewise, Ayari-Guentri et al. (2017) [28]shed light on the antioxidant potential of *H. muticus*, highlighting the remarkable potency of the stem methanolic extract, boasting an IC_50_ value of 0.541 ± 0.19 mg/mL. Conversely, the essential oil exhibited comparatively lower antioxidant activity, with an IC_50_ of 6.26 ± 0.89 mg/mL. Notably, Ayari-Guentri et al. (2017) [28] identified oxygenated monoterpenes, predominantly borneol, in the aerial parts of *H. muticus*, alongside the presence of four phenolic compounds—ferulic acid, caffeic acid, trans-cinnamic acid, and quercetin, all renowned for their antioxidant properties. Additionally, Pero et al. (2009) and Chuda et al. (1996) [29,30] have scrutinized quinic acid for its involvement in tryptophan and nicotinamide metabolism and their potent antioxidant properties, respectively. Furthermore, derivatives of guaiacol [31], cinnamic acid, and ferulic acid [32] have been acknowledged by various researchers for their significant antioxidant attributes. These collective findings highlight the diverse repertoire of antioxidant compounds present in *H. muticus* and its potential as a natural source of antioxidants.

The exploration of urease inhibition holds significant therapeutic promise, particularly in the context of combating urease-associated pathologies and infections. Urease is a key enzyme involved in the hydrolysis of urea to produce ammonia and carbon dioxide, a process implicated in various medical conditions, including urinary tract infections, kidney stones, and *Helicobacter pylori*-related gastritis and peptic ulcers [32]. By inhibiting urease activity, medicinal agents can potentially mitigate the progression of these conditions and alleviate associated symptoms. Moreover, targeting urease offers a novel approach to managing bacterial infections, as urease plays a crucial role in the survival and virulence of certain pathogenic bacteria, including *H. pylori*. Therefore, identifying natural compounds with urease-inhibiting properties, such as those found in *H. muticus* extract, not only expands our understanding of plant pharmacology but also holds promise for the development of novel therapeutic interventions against urease-related disorders and bacterial infections.

Our investigation represents a novel exploration into the anti-urease activity of *H. muticus* extract, as, to the best of our knowledge, such analysis has not been previously conducted. Thus, our study stands independent of comparisons with other studies involving the same plant species. Notably, various medicinal plants have been scrutinized for their urease-inhibiting properties, including Neem (*Azadirachta indica*), as documented by Musalia et al. (2002) [33]. The anti-urease activity observed in Neem has been attributed to the presence of diverse bioactive compounds in its leaves and seeds. Shah et al. (2014) established a significant correlation between anti-urease activity and the presence of flavonoids and phenolics [34]. Additionally, phenolic compounds such as quercetin and flavonoids have been studied for their anti-urease properties, demonstrating the ability to inhibit urease enzyme activity. Furthermore, malic acid, a naturally occurring compound found in various plants, has shown potential anti-urease activity by inhibiting the urease enzyme, as evidenced by Agha et al. (2005) [35], and Chelleng et al. (2023) [36] identified chlorogenic acid, trans-ferulic acid, and gallic acid through analytical methods such as HPLC-PDA, HR-MS, and NMR, showing notable interactions with *Helicobacter pylori* urease. Moreover, protocatechuic acid has been investigated for its in vitro activities against *H. pylori* and urease. Studies have suggested that its anti-*H. pylori* effects may operate through mechanisms such as disrupting bacterial cell membranes, inhibiting bacterial enzymes, or interfering with bacterial attachment to gastric epithelial cells, as outlined by Hassan and Švajdlenka (2017) [37].

Drug discovery is a complex and dynamic process that requires a multifaceted approach. In this study, we examine the anti-urease activity of phenolic compounds from *Hyoscyamus muticus*, focusing on their molecular interactions with urease.

Our research investigates the binding interactions of these phenolic compounds within the catalytic pocket of urease using molecular docking techniques. This analysis aims to elucidate the structural and molecular features underlying their inhibitory activity. By combining biochemical insights with computational modeling, our study seeks to deepen the understanding of the mechanisms driving urease inhibition and to identify promising candidates for novel therapeutic agents. Ultimately, this work contributes to advancing the field of anti-urease drug discovery, offering new perspectives for future research and clinical applications.

The molecular docking results, as illustrated in Table 3, shed light on the intricate interactions between *H. muticus* phenolic compounds and the urease enzyme (PDB code: 4H9M). Among the compounds studied, hyperoside emerged as the most promising, with a notable binding energy of −7.9 kcal/mol. Hyperoside exhibited strong hydrogen interactions with Asp494, Ala636, and Met588, at distances of 3.28 Å, 3.45 Å, 4.86 Å, and 2.22 Å, respectively. Additionally, hydrophobic interactions were observed with His593 and Ala440, while electrostatic interactions were prominent with Asp494.

Rutin, another significant compound, displayed a binding energy of −7.6 kcal/mol. Noteworthy hydrogen interactions were observed with Arg439, Gln635, Ala440, and Asp633, emphasizing the robust binding pattern. Hydrophobic interactions with His593, Ala636, and Met637 further contributed to the stability of the rutin–urease complex. Electrostatic interactions were particularly notable with Arg609.

Luteolin, while exhibiting a slightly lower binding energy of −6.9 kcal/mol, demonstrated hydrogen interactions with His593, Gly550, and Val519. Interestingly, this compound lacked hydrophobic interactions but engaged in electrostatic interactions with Met637 and Arg609.

Apigenin and kaempferol, with binding energies of −6.8 kcal/mol, exhibited distinct interaction profiles. Apigenin showcased hydrogen interactions with Ala440, Gly550, and Val591, coupled with hydrophobic interactions involving Met637 and His593. Kaempferol, on the other hand, displayed hydrogen interactions with Ile807, Gly562, Ly559, and Lys559, along with hydrophobic interactions with Ile563 and Lys559.

Hesperetin and chlorogenic acid, both with binding energies of −6.7 kcal/mol, presented diverse interaction patterns. Hesperetin engaged in hydrogen interactions with His593 and Arg609, while exhibiting hydrophobic interactions with Ala636, His409, His407, His545, His492, and His519. Chlorogenic acid, on the other hand, demonstrated hydrogen interactions with Ala636, Gln635, Arg439, and His593, with hydrophobic interactions primarily involving Ala636.

Lastly, rosmarinic acid displayed a binding energy of −5.8 kcal/mol, with hydrogen interactions observed with Ala436, Arg439, and His593. This compound also demonstrated a hydrophobic interaction with Ala440.

Incorporating the specific mechanisms of urease inhibition is crucial for providing a deeper understanding of the observed effects. The action of competitive inhibitors, such as the compounds in this study (e.g., hyperoside and rutin), is thought to involve binding to the active site of the urease enzyme, preventing the substrate (urea) from accessing it. In competitive inhibition, the inhibitor resembles the natural substrate, competing for the same binding site on the enzyme. As the concentration of the inhibitor increases, it effectively reduces urease activity by outcompeting urea to bind to the enzyme’s active site [38]

In the context of urease, several studies suggest that the key functional groups in inhibitors, such as hydroxyl or amide groups, play an essential role in the binding affinity, thus influencing the potency of the inhibitor. For example, inhibitors like acetohydroxamic acid (AHA) and hydroxyurea (HU) have been shown to effectively reduce urease activity through competitive inhibition [39].

Thus, the inhibition observed for hyperoside, luteolin, and rutin likely follows a similar competitive mechanism, where these compounds mimic urea and block its hydrolysis, thereby reducing ammonia production. To strengthen this conclusion, further studies focusing on binding assays and computational simulations could provide more detailed insight into the specific interactions at the enzyme’s active site.

## 4. Materials and Methods

### 4.1. Botanical Specimens

The above-ground parts of *H. muticus* were collected from the Béni-Abbès region, located 250 km southwest of Béchar and 1200 km southwest of Algiers, Algeria. After collection, the specimen was identified, air-dried, and ground into a powder. The extraction was carried out following the protocols outlined in previous studies [40,41,42,43]. Specifically, 200 g of the powdered plant material were initially macerated in petroleum ether. The residue was then progressively extracted with 600 mL of ethanol of increasing polarity. The resulting solutions were filtered under pressure using a Whatman filter paper, and the extracts were concentrated using a rotary evaporator. The final residues were used in subsequent experiments.

### 4.2. Technical Setup and Chromatography Parameters

A Shimadzu^®^ 8045 Ultra Performance Liquid Chromatography (UPLC) system from Kyoto, Japan, was utilized alongside a Shimadzu^®^ (Corporation, Kyoto, Japan) triple-quadrupole mass analyzer to evaluate the extracts. A 0.2 µm PTFE membrane was employed to filter the extracts after they had been dissolved in HPLC-grade methanol. For chromatographic separation, a Shimpack C18 reverse-phase column (2.7 µm, 2 × 150 mm) was used. Gradient elution was carried out using solvents A (water) and B (acetonitrile) at a flow rate of 0.2 mL/min. After starting at 10% B for five minutes, the elution protocol climbed to 30% B after fifteen minutes, then to 70% B after twenty-two minutes, 80% B after thirty minutes, and lastly, to 10% B after thirty-five minutes. Mass measurement was performed in negative-charge electrospray ionization (ESI) mode, with the contact degree fixed at 300 °C, the desolvation temperature adjusted to 526 °C, the cone flow of gas established at 50 L/h, and the nebulizing gas flow defined at 3 L/min [44,45,46,47,48,49].

### 4.3. Assessment of Antioxidant Effects

The detailed procedures for the various biological assay experiments are provided in the Supplementary Material. The evaluation of antioxidant potential was rigorously carried out using a wide range of methods, including superoxide alkali DMSO, β-carotene/linoleic acid, reducing power activity, cupric reducing antioxidant capacity (CUPRAC), the O-phenanthroline analytical method, the silver nanoparticle-based technique, and hydroxyl radical scavenging methods [50,51,52,53,54,55].

### 4.4. Inhibitory Capacities Targeting Urease

The enzyme solution, substrate (urea), and different inhibitor doses were added to the wells of a 96-well plate test in order to assess the urease inhibitory activity. To enable the enzyme reaction to continue, the plate was incubated. Following incubation, a detection technique such a colorimetric assay was used to determine the amount of ammonia generated, which is a sign of enzyme activity. Ammonia levels in the presence and absence of inhibitors were compared to ascertain the tested drugs’ inhibitory activity [56]. Every experiment was carried out three times. The following formula was used to obtain the percentage of inhibition:

(% inhibition) = 100 − [absorbance of extract/absorbance of control] × 100

### 4.5. Molecular Docking Analysis

The three-dimensional structure of urease (PDB ID: 4H9M) was obtained from the Protein Data Bank (PDB, www.rcsb.org), (accessed on 20 September 2024). The protein structure was optimized using UCSF Chimera 1.15 to prepare it for docking studies. Molecular docking simulations were performed for the identified phenolic compounds against urease using AutoDock 4.2, following standard protocols [57,58,59,60,61] The urease receptor was automatically prepared by adding polar hydrogen atoms and charges, while ligands were assigned Gasteiger partial charges. Non-polar hydrogens were merged during this preparation step. The ligands were treated as flexible, while the protein receptor remained rigid [62,63,64,65,66,67].

The active site of urease was identified based on information from the PDB and validated with relevant literature [68,69,70]. A grid box was created to focus on the binding pocket, with its center set at X = 25.42, Y = −50.81, Z = −27.80, and dimensions of X = 33.78, Y = 44.03, Z = 50.20. An exhaustiveness level of 8 was applied to enhance the conformational search. Docking results were evaluated based on binding energy values (in kcal/mol) and detailed analyses of hydrogen and hydrophobic interactions. Discovery Studio was used for visualization and analysis.

## 5. Conclusions

This study highlights the promising potential of *Hyoscyamus muticus* L. subsp. falezlez (Coss.) Maire as a valuable source of bioactive compounds with significant therapeutic implications. Through LC-ESI-MS/MS analysis, 21 phenolic compounds were identified, demonstrating the plant’s rich phytochemical profile. Key compounds, such as chlorogenic acid, rosmarinic acid, rutin, and p-coumaric acid, were found to be abundant, showcasing the plant’s robust antioxidant capabilities. These findings were corroborated by antioxidant assays, which confirmed the extract’s efficiency in scavenging free radicals and inhibiting lipid peroxidation.

A novel aspect of this research was the discovery of *H. muticus*’s urease inhibitory activity, with an IC_50_ value of 5.6 ± 1.20 μg/mL, positioning it as a promising candidate for conditions linked to urease activity. Molecular docking studies further revealed that key phenolic compounds, including hyperoside, rutin, and luteolin, displayed strong binding energies and favorable interactions with the urease enzyme, indicating their potential as lead compounds for drug development. This research paves the way for future exploration of *H. muticus* in medicinal and pharmaceutical sciences, offering valuable leads for further investigation and development.

## Figures and Tables

**Table 1:** Detailed analysis of phytochemical levels (μg/g extract) in *H. muticus* extract.

Analyst Number	Compound	Parent Ion (*m*/*z*)	MS2 (Collision Energy)	Chemical Formula	Type of Compound	*H. muticus* (µg dry/g Extract)
1	Quinic acid	191.085	192.085 (23), 94 (22)	C_7_H_12_O_6_	Phenolic acid	63.12 ± 22.3
2	Malic acid	133.1115	134.1115 (15), 72 (18)	C_4_H_6_O_5_	Organic acid	7.9 ± 1.3
3	tr-Aconitic acid	172.985	173.985 (13), 130 (10)	C_6_H_6_O_6_	Organic acid	N.D.
4	Gallic acid	169.1125	170.1125 (15), 80 (26)	C_7_H_6_O_5_	Phenolic acid	125.25 ± 3.4
5	Chlorogenic acid	353.0191	354.0191 (18)	C_16_H_18_O_9_	Phenolic acid	17,108.3± 1.3
6	Protocatechuic acid	153.0109	154.0109 (17), 109 (27)	C_7_H_6_O_4_	Phenolic acid	1108.3± 1.2
7	Tannic acid	183.0124	184.0124 (23), 79 (35)	C_76_H_52_O_46_	Phenolic acid	977 ± 1.3
8	tr-Caffeic acid	179.0135	180.0135 (16), 135 (25), 90 (32)	C_9_H_8_O_4_	Phenolic acid	9.3 ± 1.9
9	Vanillin	151.1136	152.1136 (18), 93 (22)	C_8_H_8_O_3_	Phenolic aldehyde	96.7 ± 1.6
10	p-Coumaric acid	163.0119	164.0119 (16), 94 (32)	C_9_H_8_O_3_	Phenolic acid	875 ± 1.3
11	Rosmarinic acid	358.9161	359.9161 (18), 134 (43)	C_18_H_16_O_8_	Phenolic acid	125.2 ± 1.1
12	Rutin	609.1300	610.1300 (38), 272 (52), 302 (39)	C_27_H_30_O_16_	Flavonoid (flavonol)	269.25 ± 1.3
13	Hesperidin	611.1303	612.1303, 466	C_28_H_34_O_15_	Flavonoid (flavanone)	N.D.
14	Hyperoside	463.1300	464.1300, 302	C_21_H_20_O_12_	Flavonoid (flavonol)	523. ± 1.7
15	4-OH Benzoic acid	137.093	138.093, 66	C_7_H_6_O_3_	Phenolic acid	N.D.
16	Salicylic acid	137.093	138.093, 66, 76	C_7_H_6_O_3_	Phenolic acid	56.3 ± 5.3
17	Myricetin	317.0179	318.0179, 152, 138	C_15_H_10_O_8_	Flavonoid (flavonol)	N.D.
18	Fisetin	285.0135	286.0135, 122	C_15_H_10_O_6_	Flavonoid (flavonol)	N.D.
19	Coumarin	147.0103	148.0103, 92, 78	C_9_H_6_O_2_	Aromatic lactone	2.3± 2.3
20	Quercetin	300.9179	301.9179, 152, 122	C_15_H_10_O_7_	Flavonoid (flavonol)	120. ± 6.3
21	Naringenin	271.0151	272.0152, 120, 108	C_15_H_12_O_5_	Flavonoid (flavanone)	N.D
22	Hesperetin	301.0164	302.0165, 137, 109	C_16_H_14_O_6_	Flavonoid (flavanone)	76. ± 2.3
23	Luteolin	285.0175	286.0176, 152, 134	C_15_H_10_O_6_	Flavonoid (flavone)	122 ± 1.02
24	Kaempferol	285.0217	286.0218, 134, 152	C_15_H_10_O_6_	Flavonoid (flavonol)	93. ± 4.2
25	Apigenin	269.0151	270.0152, 118	C_15_H_10_O_5_	Flavonoid (flavone)	563 ± 2.3
26	Rhamnetin	315.0165	316.0166, 122, 301	C_16_H_12_O_7_	Flavonoid (flavonol)	N.D.
27	Chrysin	253.0143	254.0144, 120, 108	C_15_H_10_O_4_	Flavonoid (flavone)	N.D.

Parent ion (*m*/*z*): molecular ions of the standard compounds (mass-to-charge ratio). MS2(CE): MRM fragments for the related molecular ions (CE refers to related collision energies of the fragment ions). N.D.: not detected. Values are expressed as mean ± standard deviation of five independent experiments.

**Table 2:** Antioxidant activity of *H. muticus* extract.

Assay	*H. muticus* Extract (μg/mL)	BHT (μg/mL)	BHA (μg/mL)	Ascorbic Acid (μg/mL)
CUPRAC	22.57 ± 1.2	7.75 ± 0.5	6.34 ± 0.4	7.05 ± 0.2
Reducing power	13.5 ± 2.3	/	/	6.36 ± 0.3
β-carotene	6.12 ± 1.8	9.21 ± 0.6	9.15 ± 0.4	/
DMSO alkaline	12 ± 1.2	/	/	/
SNP	6.5 ± 1.5	/	/	7.42 ± 0.1
Phenanthroline	23 ± 1.8	2.54± 0.8	2.35 ± 0.7	3.15 ± 0.5
Hydroxyl radical	39.95 ± 2.3	/	/	13.44 ± 0.7

**Table 3:** Urease inhibition by *H. muticus* extract and thiourea as a positive control.

	Urease (5 mg/mL) Inhibition (%)	C_50_ (µg/mL)
*H. muticus*	91.35	5.6 ± 1.20
*Thiourea*	96	2.6 ± 0.08

**Table 4:** Docking examination of *H. muticus*-derived phenolics with urease (4H9M).

	Binding Energy (Kcal/mol)	Detailed Examination of Hydrogen Bonds (Distance Å)	Hydrophobic Bonding	Electrostatic Bonding
Hyperoside	−7.9	Asp494 (3.28), Ala636 (3.45), Met588 (2.22)	His593, Aala440	Asp494
Rutin	−7.6	Arg439 (3.04), Arg439 (3.1) Arg439 (3.24), Gln635 (2.71), Ala440 (2.23), Asp633 (2.40) Val591 (3.78)	His593, His593, Ala636, Mer637	Arg609
Luteolin	−6.9	His593 (3.69), Gly550 (3.06), Gly550 (2.10), Val519 (3.54)	-	Met637, Arg609
Apigenine	−6.8	Ala440 (2.01), Gly550 (2.16), Val591 (3.61)	Met637, His593	Arg609
Kaemferol	−6.8	Ile807 (2.87), Gly562 (3.30), Ly559 (2.04), Lys559 (3.22)	Ile563, Lys559	-
Hesperetin	−6.7	His593 (3.33), Arg609	Ala636, His409, His407, His545, His492, His519	-
Chlorogenic acid	−6.7	Ala636 (2.04), Gln635 (2.15), Arg439 (2.44), His593 (3.15), His492 (2.35)	Ala636	Arg609
Rosmarinic acid	−5.8	Ala436 (2.40), Arg439 (3.27), His593 (3.05)	Ala440	-

## Data Availability

Data availability will be available after acceptance and publishing in this journal.

## Supplementary Materials

The following supporting information can be downloaded at: https://www.mdpi.com/article/10.3390/ijms26010370/s1.

## References

[1] Ayari-Guentri S., Saad S., Ait Kettout T., Gaceb-Terrak R., Djemouai N. (2024). Seeds of *Hyoscyamus muticus* L. Subsp. Falezlez: Morpho-anatomical Features, Phytochemical Investigation and Evidence for Antioxidant Activities. Chem. Biodivers..

[2] Ayari-Guentri S., Djemouai N., Saad S., Karoune S., Gaceb-Terrak R., Rahmania F. (2022). *Hyoscyamus muticus* L. Subsp. Falezlez Methanolic Extract: Phytochemical Composition and Biological Activities. Eur. J. Biol. Res..

[3] Almalki M.A. (2017). In Vitro Antibacterial, Antifungal and Other Medical Properties of Endangered Medicinal Plant Seeds. Pharmacol. Pharm..

[4] Ramadan M.F., Zayed R., El-Shamy H. (2007). Screening of Bioactive Lipids and Radical Scavenging Potential of Some Solanaceae Plants. Food Chem..

[5] Mohammad M.K., Almasri I.M., Tawaha K., Issa A., Al-Nadaf A., Hudaib M., AlKhatib H.S., Abu-Gharbieh E., Bustanji Y. (2010). Antioxidant, Antihyperuricemic and Xanthine Oxidase Inhibitory Activities of Hyoscyamus Reticulatus. Pharm. Biol..

[6] Lekmine S., Benslama O., Kadi K., Martín-García A.I., Yilmaz M.A., Akkal S., Boumegoura A., Alhomida A.S., Ola M.S., Ali A. (2023). LC/MS-MS Analysis of Phenolic Compounds in Hyoscyamus Albus L. Extract: In Vitro Antidiabetic Activity, in Silico Molecular Docking, and in Vivo Investigation against STZ-Induced Diabetic Mice. Pharmaceuticals.

[7] Albayrak İ., Demirci T., Baydar N.G. (2024). Enhancement of in Vitro Production of Tropane Alkaloids and Phenolic Compounds in Hyoscyamus Niger by Culture Types and Elicitor Treatments. Plant Cell Tissue Organ Cult. (PCTOC).

[8] Konieczna I., Zarnowiec P., Kwinkowski M., Kolesinska B., Fraczyk J., Kaminski Z., Kaca W. (2012). Bacterial Urease and Its Role in Long-Lasting Human Diseases. Curr. Protein Pept. Sci..

[9] Kuwahara H., Miyamoto Y., Akaike T., Kubota T., Sawa T., Okamoto S., Maeda H. (2000). Helicobacter Pylori Urease Suppresses Bactericidal Activity of Peroxynitrite via Carbon Dioxide Production. Infect. Immun..

[10] Rai R., Saraswat V.A., Dhiman R.K. (2015). Gut Microbiota: Its Role in Hepatic Encephalopathy. J. Clin. Exp. Hepatol..

[11] Sah D.K., Arjunan A., Lee B., Jung Y. (2023). Do Reactive Oxygen Species and H. Pylori Infection: A Comprehensive Review of Their Roles in Gastric Cancer Development. Antioxidants.

[12] Allam E.A.H. (2024). Urolithiasis Unveiled: Pathophysiology, Stone Dynamics, Types, and Inhibitory Mechanisms: A Review. Afr. J. Urol..

[13] Razi A., Ghiaei A., Dolatabadi F.K., Haghighi R. (2024). Unraveling the Association of Bacteria and Urinary Stones in Patients with Urolithiasis: An Update Review Article. Front Med (Lausanne).

[14] Back A., Tupper K.Y., Bai T., Chiranand P., Goldenberg F.D., Frank J.I., Brorson J.R. (2011). Ammonia-Induced Brain Swelling and Neurotoxicity in an Organotypic Slice Model. Neurol. Res..

[15] Jo D., Kim B.C., Cho K.A., Song J. (2021). The Cerebral Effect of Ammonia in Brain Aging: Blood–Brain Barrier Breakdown, Mitochondrial Dysfunction, and Neuroinflammation. J. Clin. Med..

[16] York N.E., Borofsky M.S., Lingeman J.E. (2015). Risks Associated with Drug Treatments for Kidney Stones. Expert. Opin. Drug Saf..

[17] Lafay S., Morand C., Manach C., Besson C., Scalbert A. (2006). Absorption and Metabolism of Caffeic Acid and Chlorogenic Acid in the Small Intestine of Rats. Br. J. Nutr..

[18] Rego Y.F., Queiroz M.P., Brito T.O., Carvalho P.G., de Queiroz V.T., de Fátima Â., Macedo Jr F. (2018). A Review on the Development of Urease Inhibitors as Antimicrobial Agents against Pathogenic Bacteria. J. Adv. Res..

[19] Rezvani M. (2024). Oxidative Stress-Induced Gastrointestinal Diseases: Biology and Nanomedicines—A Review. BioChem.

[20] Afzal S., Abdul Manap A.S., Attiq A., Albokhadaim I., Kandeel M., Alhojaily S.M. (2023). From Imbalance to Impairment: The Central Role of Reactive Oxygen Species in Oxidative Stress-Induced Disorders and Therapeutic Exploration. Front. Pharmacol..

[21] Čižmárová B., Hubková B., Tomečková V., Birková A. (2023). Flavonoids as Promising Natural Compounds in the Prevention and Treatment of Selected Skin Diseases. Int. J. Mol. Sci..

[22] Ismail T., Akhtar S., Sestili P., Riaz M., Ismail A., Labbe R.G. (2016). Antioxidant, Antimicrobial and Urease Inhibitory Activities of Phenolics-rich Pomegranate Peel Hydro-alcoholic Extracts. J. Food Biochem..

[23] Li L., Yang X., Tong B., Wang D., Tian X., Liu J., Chen J., Xiao X., Wang S. (2023). Rhizobacterial Compositions and Their Relationships with Soil Properties and Medicinal Bioactive Ingredients in Cinnamomum Migao. Front. Microbiol..

[24] Bilger W., Johnsen T., Schreiber U. (2001). UV-excited Chlorophyll Fluorescence as a Tool for the Assessment of UV-protection by the Epidermis of Plants. J. Exp. Bot..

[25] Ramadan R.M., Youssef F.S., Fouad E.A., Orabi A., Khalifa M.M. (2023). The Pharmacological Impact of Astragalus Membranaceus against Coccidial and Bacterial Infection in Vitro. Egypt. Pharm. J..

[26] Elsharkawy E.R., Ed-dra A., Abdallah E.M., Ali A.M.H. (2018). Antioxidant, Antimicrobial and Antifeedant Activity of Phenolic Compounds Accumulated in *Hyoscyamus muticus* L.. Afr. J. Biotechnol..

[27] Lekmine S., Boussekine S., Kadi K., Martín-García A.I., Kheddouma A., Nagaz K., Bensouici C. (2020). A Comparative Study on Chemical Profile and Biological Activities of Aerial Parts (Stems, Flowers, Leaves, Pods and Seeds) of Astragalus Gombiformis. Biocatal. Agric. Biotechnol..

[28] Ayari-Guentri S., Djemouai N., Gaceb-Terrak R., Rahmania F. (2017). Chemical Composition and Antioxidant Activity of *Hyoscyamus muticus* L. Subsp. Falezlez (Coss.) Maire from Algeria. J. Essent. Oil Bear. Plants.

[29] Pero R.W., Lund H., Leanderson T. (2009). Antioxidant Metabolism Induced by Quinic Acid. Increased Urinary Excretion of Tryptophan and Nicotinamide. Phytother. Res. Int. J. Devoted Pharmacol. Toxicol. Eval. Nat. Prod. Deriv..

[30] Chuda Y., Ono H., Ohnishi-Kameyama M., Nagata T., Tsushida T. (1996). Structural Identification of Two Antioxidant Quinic Acid Derivatives from Garland (Chrysanthemum Coronarium L.). J. Agric. Food Chem..

[31] Brand-Williams W., Cuvelier M.-E., Berset C. (1995). Use of a Free Radical Method to Evaluate Antioxidant Activity. LWT-Food Sci. Technol..

[32] Sharma P. (2011). Cinnamic Acid Derivatives: A New Chapter of Various Pharmacological Activities. J. Chem. Pharm. Res..

[33] Musalia L.M., Anandan S., Sastry V.R.B., Katiyar R.C., Agrawal D.K. (2002). Effect of Replacement of Groundnut Cake with Urea-Treated Neem (Azadirachta Indica A. Juss) Seed Kernel Cake on Nutrient Utilisation in Lambs. Asian-Australas. J. Anim. Sci..

[34] Shah N.A., Khan M.R., Sattar S., Ahmad B., Mirza B. (2014). HPLC-DAD Analysis, Antioxidant Potential and Anti-Urease Activity of Asparagus Gracilis Collected from District Islamabad. BMC Complement. Altern. Med..

[35] Agha A., Opekun A.R., Abudayyeh S., Graham D.Y. (2005). Effect of Different Organic Acids (Citric, Malic and Ascorbic) on Intragastric Urease Activity. Aliment. Pharmacol. Ther..

[36] Chelleng N., Puzari M., Chetia P., Tamuly C. (2023). Phenolic Compounds of Zanthoxylum Armatum DC as Potential Inhibitors of Urease and SARS-CoV2 Using Molecular Docking Approach and with Simulation Study. Nat. Prod. Res..

[37] Hassan S.T.S., Švajdlenka E. (2017). Biological Evaluation and Molecular Docking of Protocatechuic Acid from Hibiscus Sabdariffa L. as a Potent Urease Inhibitor by an ESI-MS Based Method. Molecules.

[38] Amtul Z., Siddiqui R.A., Choudhary M.I. (2002). Chemistry and Mechanism of Urease Inhibition. Curr. Med. Chem..

[39] Suenaga S., Takano Y., Saito T. (2023). Unraveling Binding Mechanism and Stability of Urease Inhibitors: A QM/MM MD Study. Molecules.

[40] Lekmine S., Boussekine S., Akkal S., Martín-García A.I., Boumegoura A., Kadi K., Djeghim H., Mekersi N., Bendjedid S., Bensouici C. (2021). Investigation of Photoprotective, Anti-Inflammatory, Antioxidant Capacities and LC–ESI–MS Phenolic Profile of Astragalus Gombiformis Pomel. Foods.

[41] Lekmine S., Bendjedid S., Benslama O., Martín-García A.I., Boussekine S., Kadi K., Akkal S., Nieto G., Sami R., Al-Mushhin A.A.M. (2022). Ultrasound-Assisted Extraction, LC–MS/MS Analysis, Anticholinesterase, and Antioxidant Activities of Valuable Natural Metabolites from Astragalus Armatus Willd.: In Silico Molecular Docking and In Vitro Enzymatic Studies. Antioxidants.

[42] Benslama O., Lekmine S., Mansouri N. (2023). Phytochemical Constituents of Astragalus Monspessulanus and Integrative Analysis for Its Antioxidant, Photoprotective, and Antityrosinase Activities: Experimental and Computational Investigation. Eur. J. Integr. Med..

[43] Bendjedid S., Lekmine S., Tadjine A., Djelloul R., Bensouici C. (2021). Analysis of Phytochemical Constituents, Antibacterial, Antioxidant, Photoprotective Activities and Cytotoxic Effect of Leaves Extracts and Fractions of Aloe Vera. Biocatal. Agric. Biotechnol..

[44] Morris G.M., Huey R., Lindstrom W., Sanner M.F., Belew R.K., Goodsell D.S., Olson A.J. (2009). AutoDock4 and AutoDockTools4: Automated Docking with Selective Receptor Flexibility. J. Comput. Chem..

[45] Smara M., Khalladi R., Moulai-Mostefa N., Madi K., Mansour D., Lekmine S., Benslama O., Tahraoui H., Zhang J., Amrane A. (2024). Efficiency of Hydrogen Peroxide and Fenton Reagent for Polycyclic Aromatic Hydrocarbon Degradation in Contaminated Soil: Insights from Experimental and Predictive Modeling. Processes.

[46] Fahmy N.M., Fayez S., Zengin G., Selvi S., Uba A.I., Mollica A., Bouyahya A., Ponniya S.K.M., Nilofar, Lekmine S. (2024). Chemical Exploration of Different Extracts from Phytolacca Americana Leaves and Their Potential Utilization for Global Health Problems: In Silico and Network Pharmacology Validation. J. Biomol. Struct. Dyn..

[47] Lekmine S., Benslama O., Kadi K., Ignacio Martín-García A., Shamsul Ola M., Abdullah Yilmaz M., Ali A. (2024). Therapeutic Potential of Hyoscyamus Niger-Derived Compounds: Targeting Ovarian Cancer through Antioxidant Activity and EGFR Tyrosine Kinase Inhibition. J. King Saud. Univ. Sci..

[48] Triki Z., Fergani Z., Lekmine S., Tahraoui H., Amrane A., Zamouche M., Kebir M., Assadi A.A., Khezami L., Zhang J. (2023). Numerical Modelling and Performance Evaluation of Vacuum Membrane Distillation for Energy-Efficient Seawater Desalination: Towards Energy-Efficient Solutions. Water (Basel).

[49] Lekmine S., Benslama O., Tahraoui H., Ola M.S., Laouani A., Kadi K., Martín-García A.I., Ali A. (2024). Anti-Cholinergic Effects of the Phenolic Extract from the Astragalus Crenatus Plant: A Computational and Network Pharmacology Study. Pharmaceuticals.

[50] Kunchandy E., Rao M.N.A. (1990). Oxygen Radical Scavenging Activity of Curcumin. Int. J. Pharm..

[51] Oyaizu M. (1986). Studies on Products of Browning Reaction Antioxidative Activities of Products of Browning Reaction Prepared from Glucosamine. Jpn. J. Nutr. Diet..

[52] Marco G.J. (1968). A Rapid Method for Evaluation of Antioxidants. J. Am. Oil Chem. Soc..

[53] Smirnoff N., Cumbes Q.J. (1989). Hydroxyl Radical Scavenging Activity of Compatible Solutes. Phytochemistry.

[54] Szydłowska-Czerniak A., Dianoczki C., Recseg K., Karlovits G., Szłyk E. (2008). Determination of Antioxidant Capacities of Vegetable Oils by Ferric-Ion Spectrophotometric Methods. Talanta.

[55] Özyürek M., Güngör N., Baki S., Güçlü K., Apak R. (2012). Development of a Silver Nanoparticle-Based Method for the Antioxidant Capacity Measurement of Polyphenols. Anal. Chem..

[56] Gul S., Maab S., Rafiq H., Alam A., Rehman M.U., Assad M., AlAsmari A.F., Alasmari F., Ibrahim M., Khan M. (2024). Exploring Bis-Schiff Bases with Thiobarbiturate Scaffold: In Vitro Urease Inhibition, Antioxidant Properties, and In Silico Studies. Russ. J. Bioorg Chem..

[57] Mansouri N., Benslama O., Arhab R. (2023). Homology Modeling, Docking and Molecular Dynamics Studies of Some Secondary Metabolites of Actinomycetes as Biocontrol Agents against the 3HNR Enzyme of the Phytopathogenic Fungus Alternaria Alternata. J. Biomol. Struct. Dyn..

[58] Djeghim H., Bellil I., Benslama O., Lekmine S., Temim E., Boufendi H., Postigo I., Sánchez P., Khelifi D. (2024). Effects of Genetic Diversity on the Allergenicity of Peanut (Arachis Hypogaea) Proteins: Identification of the Hypoallergenic Accessions Using BALB/c Mice Model and in Silico Analysis of Ara h 3 Allergen Cross-Reactivity. J. Proteom..

[59] Lekmine S., Benslama O., Kadi K., Brik A., Djeffali O., Ounissi M., Slimani M., Ola M.S., Eldahshan O.A., Martín-García A.I. (2024). Preliminary Investigation of Astragalus Arpilobus Subsp. Hauarensis: LC-MS/MS Chemical Profiling, In Vitro Evaluation of Antioxidant, Anti-Inflammatory Properties, Cytotoxicity, and In Silico Analysis against COX-2. Antioxidants.

[60] Boussekine S., Lekmine S., Gasmi S., Benkhedir A., Saker H., Lidoughi A. (2022). The PROTECTIVE EFFECT OF SELENIUM ON DIABETIC NEPHROPATHY IN WISTAR RATS. J. Microbiol. Biotechnol. Food Sci..

[61] Toumi S., Lekmine S., Touzout N., Moussa H., Elboughdiri N., Boudraa R., Benslama O., Kebir M., Danish S., Zhang J. (2024). Harnessing Deep Learning for Real-Time Water Quality Assessment: A Sustainable Solution. Water.

[62] Channar P.A., Saeed A., Albericio F., Larik F.A., Abbas Q., Hassan M., Raza H., Seo S.-Y. (2017). Sulfonamide-Linked Ciprofloxacin, Sulfadiazine and Amantadine Derivatives as a Novel Class of Inhibitors of Jack Bean Urease; Synthesis, Kinetic Mechanism and Molecular Docking. Molecules.

[63] Gherdaoui D., Yahoum M.M., Toumi S., Lekmine S., Lefnaoui S., Benslama O., Bouallouche R., Tahraoui H., Ola M.S., Ali A. (2024). Elucidating Chiral Resolution of Aromatic Amino Acids Using Glycopeptide Selectors: A Combined Molecular Docking and Chromatographic Study. Int. J. Mol. Sci..

[64] Moussa H., Dahmoune F., Lekmine S., Mameri A., Tahraoui H., Hamid S., Benzitoune N., Moula N., Zhang J., Amrane A. (2024). Optimization of Ultrasound-Assisted Extraction of Bioactive Compounds from Carthamus Caeruleus L. Rhizome: Integrating Central Composite Design, Gaussian Process Regression, and Multi-Objective Grey Wolf Optimization Approaches. Process Biochem..

[65] Djellal S., Dahmoune F., Aoun O., Remini H., Belbahi A., Dairi S., Moussa H., Lakehal M., Kassouar S., Lakhdari C. (2024). Optimization of Ultrasound-Assisted Extraction of Galactomannan from Carob Seeds “Ceratonia Siliqua L” and Evaluation of Their Functional Properties and in Vitro Anti-Inflammatory Activity. Sep. Sci. Technol..

[66] Hamid S., Oukil N.F., Moussa H., Mahdjoub M.M., Djihad N., Berrabah I., Bouhenna M.M., Chebrouk F., Hentabli M. (2024). Enhancing Basil Essential Oil Microencapsulation Using Pectin/Casein Biopolymers: Optimization through D-Optimal Design, Controlled Release Modeling, and Characterization. Int. J. Biol. Macromol..

[67] Moussa H., Hamid S., Mameri A., Lekmine S., Tahraoui H., Kebir M., Touzout N., Dahmoune F., Ola M.S., Zhang J. (2024). From Green Chemistry to Healthy Environments: Silver Nanoparticles as a Dual Antioxidant and Antibacterial Agents for Advancing Biomedicine and Sustainable Wastewater Treatment. Bioengineering.

[68] Rashid M., Rafique H., Roshan S., Shamas S., Iqbal Z., Ashraf Z., Abbas Q., Hassan M., Qureshi Z.U.R., Asad M.H.H. (2020). Bin Enzyme Inhibitory Kinetics and Molecular Docking Studies of Halo-Substituted Mixed Ester/Amide-Based Derivatives as Jack Bean Urease Inhibitors. Biomed. Res. Int..

[69] Yahoum M.M., Toumi S., Hentabli S., Tahraoui H., Lefnaoui S., Hadjsadok A., Amrane A., Kebir M., Moula N., Assadi A.A. (2023). Experimental Analysis and Neural Network Modeling of the Rheological Behavior of Xanthan Gum and Its Derivatives. Materials.

[70] Ben Hadj Tahar D., Triki Z., Guendouz M., Tahraoui H., Zamouche M., Kebir M., Zhang J., Amrane A. (2024). Characterization and Thermal Evaluation of a Novel Bio-Based Natural Insulation Material from Posidonia Oceanica Waste: A Sustainable Solution for Building Insulation in Algeria. ChemEngineering.

